# Nitrogen Biogeochemistry in the Caribbean Sponge, *Xestospongia muta*: A Source or Sink of Dissolved Inorganic Nitrogen?

**DOI:** 10.1371/journal.pone.0072961

**Published:** 2013-08-26

**Authors:** Cara L. Fiore, David M. Baker, Michael P. Lesser

**Affiliations:** 1 University of New Hampshire, Department of Molecular, Cellular and Biomedical Sciences, Durham, New Hampshire, United States of America; 2 Geophysical Laboratory, Carnegie Institute of Washington, Washington, District of Columbia, United States of America; Missouri University of Science and Technology, United States of America

## Abstract

**Background:**

Sponges have long been known to be ecologically important members of the benthic fauna on coral reefs. Recently, it has been shown that sponges are also important contributors to the nitrogen biogeochemistry of coral reefs. The studies that have been done show that most sponges are net sources of dissolved inorganic nitrogen (DIN; NH_4_^+^ and NO_3_^−^) and that nitrification, mediated by their symbiotic prokaryotes, is the primary process involved in supplying DIN to adjacent reefs.

**Methodology/Principal Findings:**

A natural experiment was conducted with the Caribbean sponge *Xestospongia muta* from three different locations (Florida Keys, USA; Lee Stocking Island, Bahamas and Little Cayman, Cayman Islands). The DIN fluxes of sponges were studied using nutrient analysis, stable isotope ratios, and isotope tracer experiments. Results showed that the fluxes of DIN were variable between locations and that *X. muta* can be either a source or sink of DIN. Stable isotope values of sponge and symbiotic bacterial fractions indicate that the prokaryotic community is capable of taking up both NH_4_^+^ and NO_3_^−^ while the differences in *δ* ^15^N between the sponge and bacterial fractions from the NH_4_^+^ tracer experiment suggest that there is translocation of labeled N from the symbiotic bacteria to the host.

**Conclusions/Significance:**

Nitrogen cycling in *X. muta* appears to be more complex than previous studies have shown and our results suggest that anaerobic processes such as denitrification or anammox occur in these sponges in addition to aerobic nitrification. Furthermore, the metabolism of this sponge and its prokaryotic symbionts may have a significant impact on the nitrogen biogeochemistry on Caribbean coral reefs by releasing large amounts of DIN, including higher NH_4_^+^ concentrations that previously reported.

## Introduction

Sponges are an ecologically dominant component in many marine ecosystems, including coral reefs, where they contribute to the consolidation of reefs, prevent erosion, filter large quantities of seawater and provide habitat and food for many invertebrates and fishes [1]–[3]. Because of their ability to efficiently filter picoplankton, sponges can also contribute significantly to the coupling of productivity in the overlying water column to the benthos [1], [4], [5]. More recently, sponges and their prokaryotic symbionts have become an important area of research to quantify the fluxes of DIN by sponges and the biogeochemical cycling of nutrients on coral reefs [4], [6]–[8].

Nitrogen cycling on tropical coral reefs is particularly important, as nitrogen is a limiting nutrient and the success of several coral reef taxa (e.g., corals) is dependent on their symbiotic partners and the efficient re-cycling of nitrogen between host and symbionts [9]. Compared to ambient seawater the excurrent water of actively pumping sponges is often enriched in DIN such as NO_3_^−^ (or NO_2_^−^+NO_3_^−^) as a result of nitrification [10]. This has been documented for sponges from coral reefs, mangroves, seagrass beds [7], [8], [11]–[13], as well as sponges from temperate and cold-water environments [14]–[16]. In fact, coral reef sponges have been documented to have rates of nitrification that are significantly higher (5.8–16.0 mmol m^−2^ d^−1^ NO_3_^−^) [7], [13] than what has been reported for benthic habitats such as microbial mats (up to 1.4 mmol m^−2^ d^−1^ NO_3_^−^ [17]) or coral reef sediment (1.7 mmol m^−2^ d^−1^ NO_3_^−^ [18]).

All pathways of nitrogen biogeochemistry have been reported to occur in sponges [10] including nitrogen fixation which was initially measured using the acetylene reduction method [19]–[21]. Stable isotope tracer studies (i.e., ^15^N_2_) later confirmed the presence of nitrogen fixation, albeit at low rates, in several species of sponges from coral reefs [21], [22]. Recently, the first sponge-derived *nif*H gene sequences and transcripts, which encode for the iron protein component of the nitrogenase enzyme responsible for nitrogen fixation, were documented in two sponge species from the Florida Keys [23]. *nif*H genes have also been recovered from *Xestospongia muta* from multiple locations in the Caribbean [Fiore and Lesser unpublished] and hypoxic/anoxic conditions, known to occur in sponges [10], would be required for activity since nitrogenase is inactivated by molecular oxygen and only fixes nitrogen under anaerobic or micro-aerobic conditions [10]. With the presence of anaerobic microhabitats in sponges [10], [24], other anaerobic nitrogen transformations including sulfate reduction, denitrification and anaerobic ammonium oxidation (anammox) have been observed and quantified using stable isotopic tracer methods, radiolabeled isotopes and recovery of gene specific sequences for key enzymes [16], [24], [25]. Interestingly, genomic analysis of the candidate phylum Poribacteria, which is found in sponges from numerous marine habitats [26], [27], suggests that Poribacteria may also be capable of denitrification [28].

Nitrification in sponges, which produces the bulk of the DIN released [8], [13], has been documented using collection and incubation methods combined with nutrient analyses [12], [13], [16]. Southwell et al. [7], [8] was the first to use an *in situ* method to identify actively nitrifying sponges and estimate the flux of DIN onto the adjacent coral reef, and found no significant difference in flux of DIN between the incubation and in situ methods. The interest in nutrient fluxes mediated by sponges and their symbionts as well as the nutrient biogeochemistry of coral reefs has resulted in a surge of research into the prokaryotic community composition of sponges and the processes they mediate. Recently, this has included the use of high throughput sequencing methods, such as 454 pyrosequencing of the 16S rRNA gene [[27], [29], [30],Fiore and Lesser unpublished] which have increased our understanding of the prokaryotic composition of many sponge species from different marine habitats. This genetic information has provided useful complementary insight for characterizing prokaryotic mediated nutrient cycling in these sponges.

On Caribbean coral reefs *Xestospongia muta* is an ecologically dominant member of the benthic community, and on Conch Reef, FL (USA) the number of *X. muta* has been shown to be significantly increasing over time [31]. *Xestospongia muta* is also characterized as a high microbial abundance sponge [32] but little was known about the composition of this community other than it contained Cyanobacteria [33] until recent studies documented a diverse prokaryotic community in *Xestospongia muta* and other members of this genus [[34], [35],Fiore and Lesser unpublished]. Additionally, *X. muta* outside of the Florida Keys have not been as well studied generally with quantitative 454 pyrosequencing comparing the prokaryotic symbionts of *X. muta* from the Florida Keys, Cayman Islands and Bahamas having been recently completed [Fiore and Lesser unpublished].

The primary goal of this study was to quantify DIN fluxes in *X. muta* from the same three populations where our 16S rRNA 454 pyrosequencing study was done. We ask whether sponges from these same populations in the Caribbean have different fluxes of DIN, and potentially how any differences in DIN fluxes, may be related to the taxonomy of their symbiotic prokaryotes using a comparative approach and a natural experiment [36].

## Materials and Methods

### Sample Locations

Replicate sponges (n = 6) were sampled at approximately 15 m depth between 9 and 10 AM and again between 4 and 5 PM when indicated from each of three locations: Rock Bottom Reef, Little Cayman, Cayman Islands (LC) (19°42′7.36″ N, 80°3′24.94″ W), North Perry Reef, Lee Stocking Island, Bahamas (LSI) (23°47′0.03″ N, 76°6′5.14″ W), and Conch Reef, Key Largo, FL (FL) (24°57′0.03″ N, 80°27′11.16″ W). All populations were sampled during the late spring and early summer of 2011 where the maximum irradiance of photosynthetically active radiation (PAR; 400–700 nm) at noon for all three locations is ∼500–600 µmol quanta m^−2^ s^−1^ [Lesser unpublished]. Necessary permits were obtained for all three locations: the Marine Conservation Board, Cayman Islands; Department of Marine Resources, Bahamas; NOAA ONMS permit number FKNMS-2011-066 for Conch Reef, Florida Keys.

### Nutrient analyses and rates of sponge pumping

Ambient and excurrent water samples for nutrient analysis were collected from individual sponges (n = 6) at each location for nutrient analysis by slowly filling 100 ml syringes and placing all water samples on ice for transport to shore. Ambient water was obtained by filling the syringe adjacent to each sponge (within 20 cm of the sponge body wall) and excurrent water was obtained by placing weighted Tygon ® tubing inside the sponge close to the base of the spongocoel that was attached to a 100 ml syringe and drawing water into the syringe slowly (∼1 ml s^−1^). Syringes were then purged of approximately 10 ml and then 40 ml was saved and frozen for NH_4_^+^ and NO_2_^−^+NO_3_^−^ analysis (NO_x_^−^). For the nutrient analyses the water samples were thawed and filtered (0.22 µm, Whatman, USA) to remove particulate matter then re-frozen and sent to the Nutrient Analytical Facility at Woods Hole Oceanographic Institute (WHOI, Woods Hole, MA, USA) for analysis using a Lachet QuickChem 8000 (flow injection analysis system) according to standard protocols to determine concentration of NH_4_^+^ and NO_x_^−^. Instrumental errors associated with the measurements were calculated as relative standard deviation (RSD) and includes: NH_4_^+^ −0.6% measured RSD, and NO_2_^−^+NO_3_^−^ −0.59% measured RSD. Sponges were marked near their base with labeled flagging tape attached to nails embedded in the substrate to facilitate repeated measurements on the same sponges.

The volume flow or pumping rates for each individual sponge was determined as previously described [37]. A small amount (∼1 ml) of fluorescein dye was injected using a syringe and 16 gauge needle into the sponge just below the base of the spongocoel and the time(s) that the dye front took from its first appearance at the base of the spongocoel to the top of the spongocoel was recorded to obtain the centerline fluid velocity to calculate volume flux or pumping rate. We understand that unlike previous studies on tubular sponges where plug flow can be reasonably assumed (e.g., [37]) the morphology of *X. muta* likely creates more complicated excurrent plumes where the velocity across the osculum is not uniform [38]. This is easily observed using the timing of multiple dye tracks on *X. muta* injected in different locations with dye tracks closer to the sponge wall being slower than the centerline flow [Lesser unpublished]. As a result we recognize that our measurements of volume flow or pumping rates are likely to be an overestimate. That said our estimates of volume flow are in agreement with the results of Southwell et al. [8] using similar techniques for *X. muta*. Additionally, in our hands we have never observed cessation of pumping, or other artifacts, as a result of exposure to fluorescein in both thin walled and thick walled sponges [5], [37]. Both spongocoel and total sponge volume were calculated by measuring sponge height, base circumference, osculum diameter, and spongocoel depth and inner diameter with a measuring tape (to ±1.0 mm) and volume calculated as previously described [31]. The mass (kg) of individual sponges was then calculated by multiplying the individual total sponge volume (l), obtained as described above, by the average density of *X. muta* sponges (0.617 g cm^−3^) which was determined from direct measurements of the displacement volume and mass of pieces (n = 5) of sponge (including both mesohyl and pinacoderm). The flux of nutrients was then calculated by multiplying the ΔDIN (the difference in nutrient concentration between the ambient and excurrent water in *µ*mol l^−1^ by the flow rate (cm s^−1^) and normalized to both sponge volume and mass for comparisons between sites.

Pumping rates and nutrient flux data were tested for assumptions of ANOVA and if the data failed either normality or homoscedasticity a constant integer to all values was added followed by log transformation. The transformed data passed Bartlett's test [39] for homoscedasticity but often failed the Shapiro-Wilks [40] test for normality. Because Bartlett's test is sensitive to deviations in normality [41] we choose to proceed with ANOVA, which is known to be robust to deviations from normality [42], on the transformed data. To determine if time of day was a significant factor in the flux of DIN, a two-way ANOVA with interaction was performed using the statistical program R [43] with time (AM and PM) and location as fixed factors for the flux of NO_x_^−^, NH_4_^+^, total DIN and pumping rate as the response variables. A repeated measures ANOVA was not performed because the general requirement of this approach is three time points. Since the effect of time and interaction of time with location was not significant the flux of NO_x_^−^, NH_4_^+^, total DIN and pumping rate, ΔDIN and pumping rates for each location were calculated by averaging the AM and PM values for each individual sponge. Collapsing the design to a single factor analysis to examine differences between locations was then assessed using a one-way ANOVA with location as a fixed factor [41].

### Flow Cytometry

Ambient and excurrent water samples were collected as described above for the nutrient analyses for another set of sponges (n = 4) from LSI only. Approximately 3 ml from each collected water sample were fixed in electron microscopy grade paraformaldehyde at a final concentration of 0.5% in filtered (0.22 µm) seawater and frozen at −50°C. Frozen water samples were sent to the Bigelow Laboratory for Ocean Sciences J.J. MacIsaac Aquatic Cytometry Facility where they were stored in liquid nitrogen until analysis. Each sample was analyzed for cell abundances using a Becton Dickinson FACScan flow cytometer with a 30 mW, 488 nm laser. Simultaneous measurements of forward light scattering (FSC, relative size), 90° light scatter (SSC), chlorophyll fluorescence (>650 nm), and phycoerythrin fluorescence (560–590 nm) were made simultaneously on each sample as previously described [5]. Calculations of cyanobacteria, prochlorophyte, and heterotrophic cell concentration and filtering efficiency were performed as previously described [5]. Technical replicates (n = 2) were averaged for each sample and the cell abundance of heterotrophic bacteria was determined using PicoGreen (Molecular Probes), a dsDNA specific dye, which stains all prokaryotes (emission fluorescence 515–525 nm). Subtraction of the chl *a* containing picoplankton from the total prokaryotes yielded the heterotrophic bacterial component of the community while cyanobacterial and prochlorophyte cells were differentiated by the presence or absence, respectively, of phycoerythrin fluorescence. All filtered cells were converted to carbon and nitrogen equivalents using the following conversions; heterotrophic bacteria: 20 fg C cell^−1^ [44], *Prochlorococcus*: 53 fg C cell^−1^ [45], *Synechococcus*: 470 fg C cell^−1^ [46], heterotrophic bacteria: 3.3 fg N cell^−1^ [47], *Prochlorococcus*: 9.4 fg N cell^−1^ [48], *Synechococcus*: 35 fg N cell^−1^ [48]. Data were log transformed or arcsin transformed as necessary and an ANOVA followed by Tukey's HSD were performed to test for significant differences in the number of filtered cells between cell types (cyanobacteria, prochlorophytes, heterotrophic bacteria and total cells), filtration efficiency and total particulate carbon (POC) and nitrogen (PON) consumed by sponges.

### Stable isotopic analyses and tracer experiments

Sponge samples that were frozen without buffer (n = 3 each location) were later lyophilized, ground to a powder with a mortar and pestle, and then acid treated with 1 M HCl to remove carbonate and rinsed with distilled water and allowed to dry. An analysis of samples from FL, separated into the outer pigmented layers of the sponge and the non-pigmented inner tissues (containing the pinacoderm and outer mesohyl respectively), showed no significant differences in stable isotope signatures [Fiore and Lesser, unpublished data] so whole cross-sections of sponge samples, consisting of both pinacoderm and mesohyl, from all locations were analyzed. Samples were then sent to the Marine Biological Laboratory (MBL) for the analysis of particulate C and N as well as the natural abundance of the stable isotopes *δ* ^15^N and *δ* ^13^C. Samples were analyzed using a Europa ANCA-SL elemental analyzer-gas chromatograph attached to a continuous-flow Europa 20–20 gas source stable isotope ratio mass spectrometer. The carbon isotope results are reported relative to Vienna Pee Dee Belemnite and nitrogen isotope results are reported relative to atmospheric air and both are expressed using the delta (*δ*) notation in units per mil (‰). The analytical precision of the instrument is ±0.1‰, and the mean precision of sample replicates for *δ* ^13^C was ±0.4‰ and *δ* ^15^N was ±0.2‰. A one-way ANOVA was used to test for significant differences between locations for *δ* ^13^C, *δ* ^15^N and C∶N ratios followed by the post hoc multiple comparison Tukey's HSD test as needed.

Two stable isotope tracer experiments were conducted during the summer of 2011 at LSI: the first used Na^15^NO_3_ (5 mg l^−1^ final concentration) plus H^13^CO_3_ (50 mg l^−1^) and the second used ^15^NH_4_ (0.31 mg l^−1^) as tracers (Sigma-Aldrich, USA). The method was the same for each experiment: 11 individual *X. muta* (average volume 172±77 ml or mass 0.106±0.048 kg; mean ±SD) were collected by cutting through the bottom of the sponge but keeping the tissue from the base of the spongocoel intact, from approximately 12 m at North and South Perry reefs at LSI and held in a large holding tank with flow through seawater for 5 d to recover from being removed from the reef. Care was taken to ensure that sponges were never exposed to air and that light levels were maintained at the same levels as found at ∼12 m using neutral density screens over the outdoor flowing seawater tanks. Sponges were checked for pumping activity using fluorescein dye and incubated statically with the tracer compound(s) for 4 h. Subsequently, T_0_ sponges (n = 3) were then removed and stored for analysis.

The remaining sponges were placed in individual aquaria with flow through seawater and sponges were sampled at 3 h (n = 2), 6 h (n = 3) and at 12 h (n = 3) for each fraction. Frozen samples were initially processed by separating the bacteria and sponge fractions following the methods of Freeman and Thacker [49] and Freeman et al. [50] except for two steps: an initial centrifugation was performed at 520× g for 4 min, and the resulting sponge pellet was rinsed an additional two times. The purity of the sponge and bacterial fractions were assessed using light and epifluorescence light microscopy as described by Freeman and Thacker [49]. The sponge fractions always contained large cells (8–10 µm diameter) consisting of at least 85% per microscopic field and exhibiting low natural fluorescence, whereas the bacterial fractions contained only small cells (<1–2 µm diameter) and high natural fluorescence. While efforts were made to separate and purify the sponge fraction as much as possible from all prokaryotic cells, it is possible that some prokaryotes that were located intracellularly were not detected (non-fluorescent) in the sponge fraction. These methods have been shown to be effective for other sponge species [49] and were optimized for use with *X. muta*. Additionally, a one-way ANOVA of the C∶N ratios for the two fractions yielded significant differences (F_1,13_ = 8.38, p = 0.01 (NH_4_^+^ tracer experiment); F_1,13_ = 24.6, p<0.01 (NO_3_^−^+HCO_3_^−^ tracer experiment) indicating that good separation of these fractions occurred. It is likely, however, that some contamination occurred and was considered when interpreting the results of these experiments. Samples were then lyophilized and ∼1.0 mg was weighed and placed into silver capsules (Costech, CA, USA) and acidified three times with 20 *µ*l of 12 M HCl. Samples were allowed to dry in between acidifications, then oven dried at 50°C for 48 h. Samples were combusted in a Carlo-Erba NC2500 elemental analyzer, and the resulting gas was analyzed in a Thermo Delta V isotope ratio mass spectrometer via a Conflo III open-split interface. The analytical precision of the instrument was ±0.2‰, and the mean standard deviation of sample replicates for *δ* ^13^C was ±0.4‰ and for *δ* ^15^N it was ±0.8‰ for enriched samples and ±0.1‰ and ±0.1‰ for natural abundance samples, respectively. For the tracer experiments the data were log transformed as necessary to meet the assumptions of parametric statistics and a two-way ANOVA with interaction, with fraction and time as fixed factors, was used to assess treatment effects.

## Results

### Stable Isotopic Signatures of Sponges

**Figure 1:**
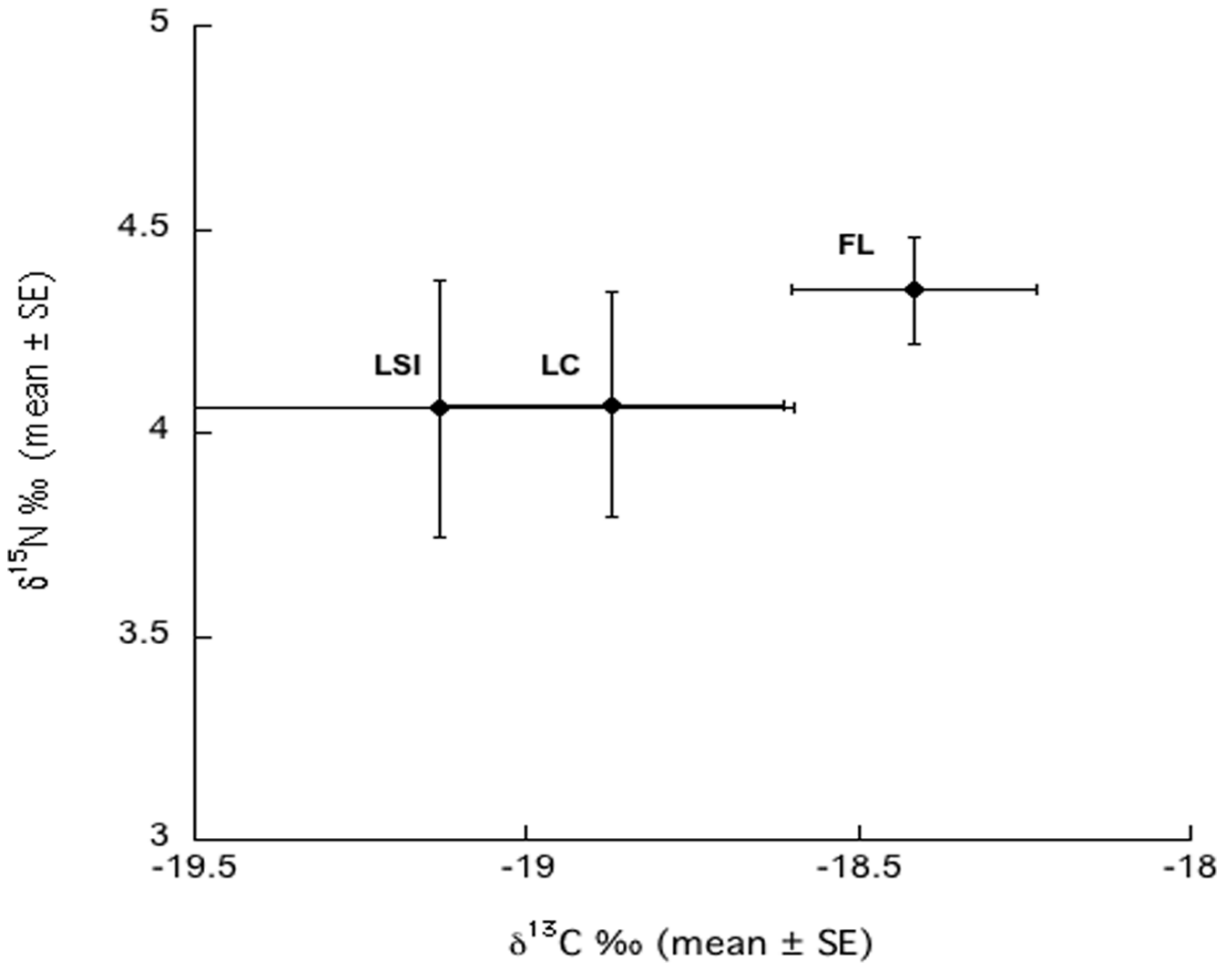
δ^15^N and δ^13^C values (mean ±SE) for *Xestospongia muta* (n = 3) for each location. FL =  Florida Keys LC = Little Cayman, LSI = Lee Stocking Island, Bahamas.

The values of *δ* ^13^C from each location were not significantly different from each other (ANOVA, F_2,6_ = 1.16, p = 0.38). The *δ* ^13^C of sponge samples ranged from −19.1 to −18.4‰ (Fig. 1). The *δ* ^15^N of sponge samples ranged from 4.0–4.4‰ (Fig. 1), and were not significantly different between locations (ANOVA, F_2,6_ = 0.52, p = 0.62). The ratios of C∶N were significantly different between locations (ANOVA, F_2,6_ = 22.57, p = 0.002), with post hoc pairwise comparisons showing that FL sponges had significantly higher C∶N ratios than LSI (Tukey's HSD, p<0.05), and that LC sponges significantly higher than LSI (Tukey's HSD, p<0.05). There was no significant difference between LC and FL (Tukey's HSD, p>0.05). Despite the significant results for C∶N ratios the mean values did not vary greatly, with a range of 4.33–4.83.

### Inorganic Nitrogen Fluxes in *Xestospongia muta*

The difference in nutrient concentration between the ambient and excurrent of NH_4_^+^, NO_3_^−^+NO_2_^−^ (NO_x_^−^) and total DIN for *X. muta* varied considerably between individual sponges as expected for sponges over a large size range (Table 1). The ΔNH_4_^+^ values were not significantly different between locations (ANOVA, F_2,15_ = 3.19, p = 0.07) as were the ΔNO_x_^−^ values (ANOVA, F_2,15_ = 2.58, p = 0.11). ΔDIN values, however, were significantly different between sites (ANOVA, F_2,15_ = 6.82, p = 0.008) with post hoc pairwise comparisons showing that FL sponges were significantly lower than both LSI and LC sponges LSI (Tukey's HSD, p<0.05) which were not significantly different than each other (Tukey's HSD, p>0.05). No measurements of ambient NO_x_^−^ exceeded 4 µM eliminating the potential for ambient nutrient concentrations to be confounded by oceanographic features such as internal waves [51], [52]. Sponge pumping rates varied with size (Table 1) and did not differ significantly with location (ANOVA, F_2, 15_ = 1.61, p = 0.23).

**Table 1:** Calculated volume, mass, and flux parameters for samples of *Xestospongia muta* at each location.

Location	Spongocoel (L)	Volume (L)	Mass (kg)	Flow rate (L h-1)	ΔDIN NH_4_^+^ (µmol L^−1^)	ΔDIN NO_x_ (µmol L^−1^)	Flux NH_4_^+^ (µmol h^−1^ L^−1^)	Flux NO_x_ (µmol h^−1^ L^−1^)	Flux DIN (µmol h^−1^ L^−1^)	Flux NH_4_^+^ (µmol h^−1^ kg^−1^)	Flux NO_x_ (µmol h^−1^ kg^−1^)	Flux DIN (µmol h^−1^ kg^−1^)
FL	4	43	26.8	4050	−0.15	−0.99	−13	−103	−116	−21	−167	−188
FL	20	101	62.2	18090	0.00	0.65	0	117	117	0	189	189
FL	39	36	22.4	62370	0.35	−0.30	668	−496	172	904	−804	100
FL	33	111	68.2	21938	0.10	−0.09	26	−26	1	43	−42	1
FL	14	76	47.0	15377	0.90	0.26	−188	51	−137	−387	82	−305
FL	7	37	22.9	6480	−0.05	−0.01	−9	−2	−9	−5	−3	−7
LC	12	64	39.3	4656	5.10	2.60	328	116	444	1653	1037	2690
LC	5	12	7.4	2649	1.80	−0.30	2665	−181	2484	1313	−122	1191
LC	4	50	31.1	5423	0.35	0.90	38	92	130	681	1887	2567
LC	6	49	30.3	7109	0.40	0.35	81	71	151	333	1021	1354
LC	4	17	10.5	2145	3.30	0.25	213	41	254	4279	324	4603
LC	14	63	38.9	8463	0.45	0.20	60	31	90	350	272	622
LSI	5	22	13.6	6912	0.36	1.23	−180	438	258	−292	710	418
LSI	10	45	27.6	16640	0.11	0.53	40	66	106	64	107	172
LSI	29	81	50.2	28800	0.50	0.63	213	159	372	344	258	603
LSI	2	18	11.0	11040	−0.12	0.87	−162	1202	1040	−262	1948	1686
LSI	3	23	14.0	2687	−0.19	0.30	12	15	27	19	24	43
LSI	2	14	8.6	11220	2.53	0.67	4313	793	5106	6990	1286	8276

**Figure 2:**
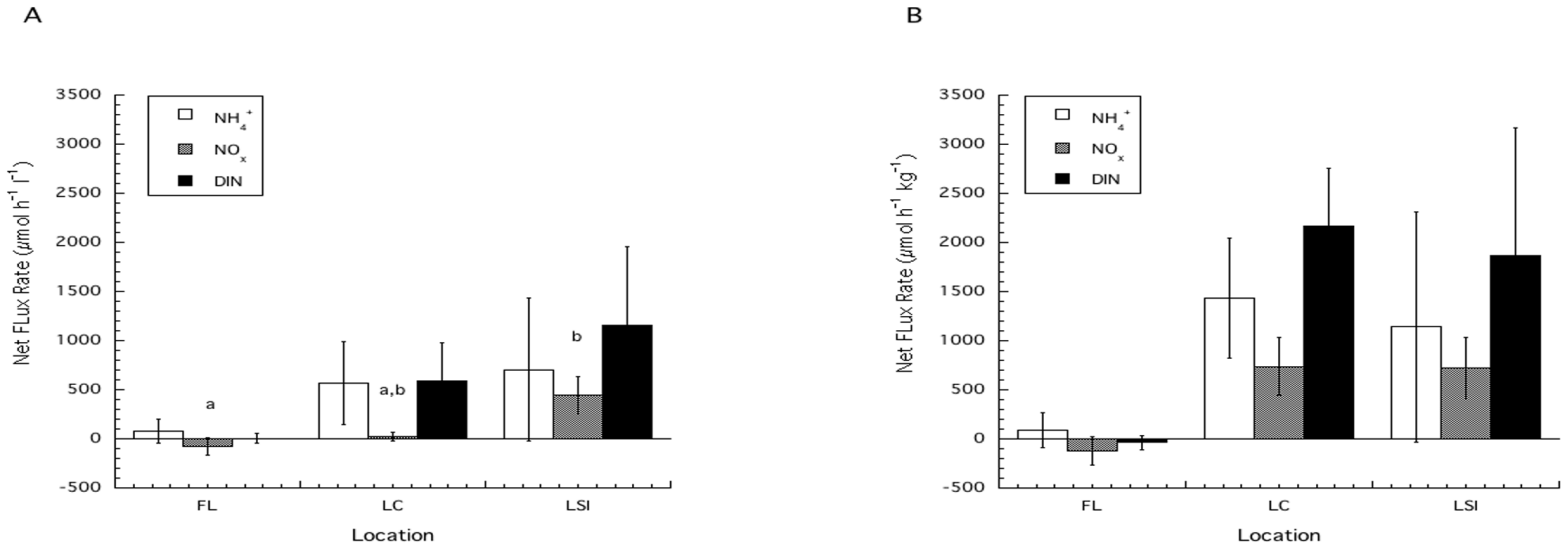
The average flux (mean ±SE) of NH_4_^+^, NO_x_^−^ and DIN. The average flux is shown for each location normalized to sponge volume (A) and mass (B). Treatment groups with similar superscripts are not statistically different from each other.

The volume and mass normalized fluxes of NH_4_^+^ (Table 1, Fig. 2 a) were not significantly different between locations (ANOVA, F_2, 15_ = 0.45, p = 0.65 (volume); F_2, 15_ = 0.85, p = 0.45 (mass)). The fluxes of NO_x_^−^ normalized to sponge volume (Table 1, Fig. 2 b) were significantly different between locations (ANOVA, F_2, 15_ = 4.89, p = 0.02) with FL sponges significantly lower than LSI and LC not significantly different than either FL or LSI (Fig. 2A) but when normalized to mass did not show a significant effect of location (ANOVA, F_2, 15_ = 3.56, p = 0.054). The flux of total DIN (NO_x_^−^+NH_4_^+^) normalized to volume and to mass were not significantly different among locations (ANOVA, F_2, 15_ = 1.24, p = 0.32 (volume); F_2, 15_ = 2.09, p = 0.16 (mass)).

### Feeding Study

**Figure 3:**
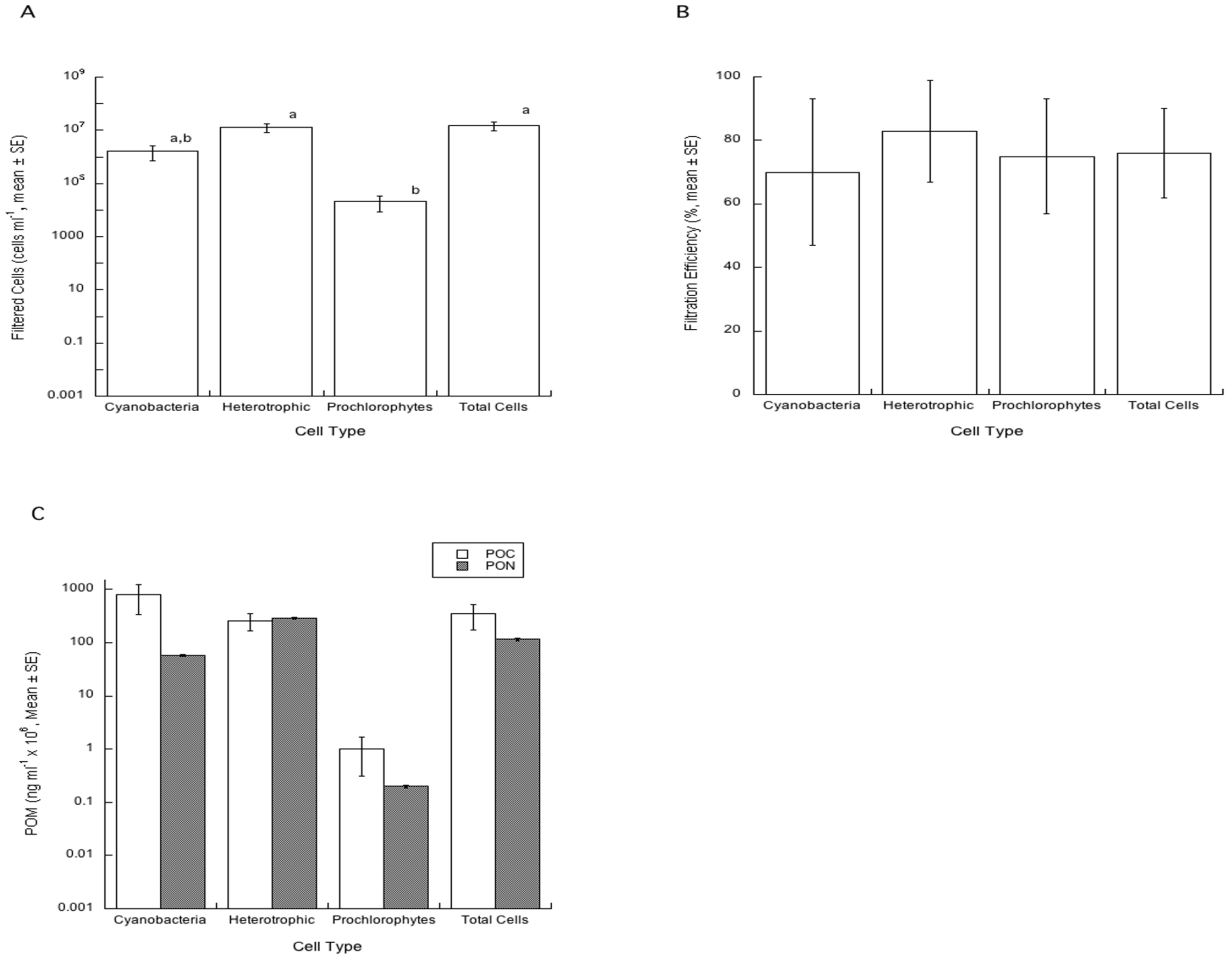
Filtration of bacterioplankton from the water column by *X.* *muta*. Number of filtered cells (A), filtration efficiency (B), and particulate organic matter as carbon and nitrogen (C) available from filtered cells for *X. muta* from LSI (n = 4). Treatment groups (mean ±SE) with similar superscripts are not statistically different from each other.

*Xestospongia muta* (n = 4) from LSI instantaneously filtered an average of 1.5×10^7^ cells ml^−1^ and there was a significant effect of cell type (ANOVA, F_3, 12_ = 4.45, p = 0.03) (Fig. 3A). The number of both total cells and heterotrophic bacteria filtered was significantly higher than that of prochlorophytes (Tukey's HSD, p<0.05) but not cyanobacteria (Tukey's HSD, p>0.05), while the number of cyanobacterial cells filtered was indistinguishable (Tukey's HSD, p>0.05) from the prochlorophyte or total cell and heterotrophic cell groupings (Fig. 3A). For the filtration efficiency of each cell type there were no significant differences (ANOVA, F_3, 12_ = 0.45, p = 0.72) (Fig. 3B). The total amount of POC for each retained cell type was greatest for cyanoacteria, but there was no significant difference between cell types (ANOVA, F_3, 12_ = 2.77, p = 0.09). Differences between the amount of PON for each retained cell type was significant (ANOVA, F_3, 12_ = 4.68, p = 0.02) and greatest for total cells and heterotrophic bacteria compared to prochlorophytes (Tukey's HSD, p = 0.03) but not cyanobacteria (Tukey's HSD, p>0.05), while the PON of cyanobacterial cells was indistinguishable (Tukey's HSD, p>0.05) from the prochlorophyte or total cell and heterotrophic cell groupings (Fig. 3C).

### Nitrogen tracer experiment: Nitrate and Bicarbonate

**Figure 4:**
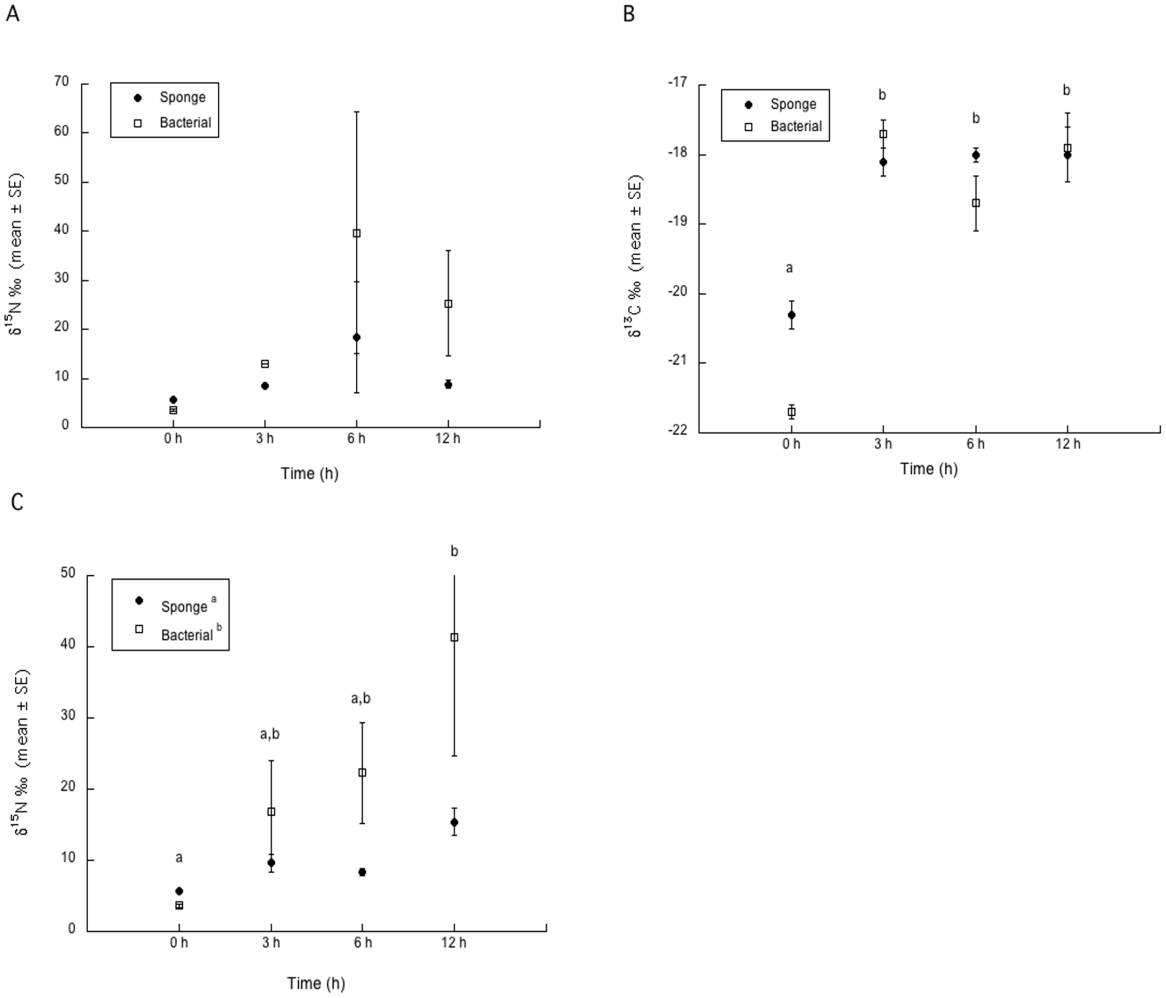
*δ* ^15^N and *δ* ^13^C isotopic values from uptake experiments with *X.* *muta*. *δ* ^15^N over time (A) for ^15^N nitrate and *δ* ^13C^ over time (B) for ^13^C bicarbonate enriched sponge and bacterial fractions, and *δ* ^15^N over time for ^15^N ammonium enriched sponge and bacterial fractions (C). Samples collected at 3 h were under low irradiances while samples collected at 6 h had been exposed to sunlight (Under neutral density screens representative of irradiances at the depth of collection) and samples from 12 h were collected at night (for both experiments). Treatment groups (mean ±SE) with similar superscripts are not statistically different from each other.

While sponge and bacterial fractions became more enriched from 3 to 6 hours there was no significant effect of enrichment of ^15^N from the NO_3_^−^ tracer in those fractions (ANOVA, F_7, 14_ = 0.84, p = 0.57). There was also no significant difference between sponge and bacterial fractions or over time or the interaction of fraction and time (fraction, F_1,3_ = 0.89, p = 0.36; time, F_1,3_ = 1.16, p = 0.36; interaction term, F_1,3_ = 0.28, p = 0.84) (Fig. 4A). Additionally, the enrichment of ^13^C from the bicarbonate tracer experiment was significant (ANOVA, F_7, 14_ = 20.9, p<0.0001) with fraction being non-significant (F_1,3_ = 0.13, p = 0.13) and time being significant (F_1,3_ = 44.3, p<0.001) with a non-significant interaction term, (F_1,3_ = 3.2, p = 0.056) (Fig. 4B). As a result post-hoc multiple comparison tests were only performed for time. Both the sponge and bacterial fractions became more enriched in^13^C then the sponge fraction over time with all sampling periods being significantly different than T_0_ (Tukey's HSD p<0.05) and not significantly different (Tukeys' HSD p>0.05) from each other (Fig. 4B).

### Nitrogen tracer experiment: Ammonium

There was significant enrichment of ^15^N from the NH_4_^+^ tracer in the experimental sponges (ANOVA, F_7, 13_ = 3.38, p = 0.03) with the effects tests for fraction (F_1,3_ = 6.4, p = 0.03) and time (F_1,3_ = 5.0, p = 0.02) being significant and the interaction term non-significant (F_1,3_ = 1.8, p = 0.19) (Fig. 4C). Post-hoc multiple comparison testing for time revealed a significant (Tukey's HSD p<0.05) increase in enrichment over time with the bacterial fraction exhibiting greater enrichment (Fig. 4C).

## Discussion

This study of the inorganic nitrogen fluxes in *Xestospongia muta* is the first to show differences in net fluxes of DIN both within and between populations of this ecologically dominant sponge on Caribbean coral reefs. *Xestospongia muta* densities are increasing [31], so quantifying both the fluxes of DIN and understanding the underlying processes driving the nitrogen biogeochemistry in this sponge is important for understanding the DIN availability on reefs in the Caribbean.

In a survey of nitrification in sponges on Conch Reef, Florida Southwell et al. [7], [8] reported evidence of nitrification in nine out of twelve sponge species, including *X. muta*. The rates of nitrification measured in these sponges varied, but overall they were at least two orders of magnitude higher than other habitats (e.g., benthos, coral rubble). In the current study, we observed similar rates of *X. muta* pumping activity reported for Conch Reef sponges by Southwell et al. [8]. However, unlike Southwell et al. [7], [8], *X. muta* from Conch Reef exhibited a negative flux of NO_x_^−^, indicating that either denitrification or anammox processes were taking place or possibly dissimilatory nitrate reduction. Our results from LSI and LC are consistent with Southwell et al. [7], [8] and for LSI the fluxes of NO_x_^−^ are significantly higher than fluxes of NO_x_^−^ from FL compared to Southwell et al. [8].

*Xestopongia muta* were actively pumping for all measurements taken during this study, which was not significantly different over time of day or between locations. While pumping rates were not significantly different, the observed variability in the unidirectional pumping of sponges has the potential to create microhabitats where both anaerobic nitrogen transformations (e.g., denitrification) and aerobic nitrogen transformations (e.g., nitrification) could occur [10], [16], [24], [53]. Additionally, recent studies of the bacterial communities of *X. muta* using16S rRNA sequencing [35,Fiore and Lesser unpublished] have reported many bacterial groups that are capable of denitrification and anammox (i.e., Burkholderiales, Pseudoalteromonadaceae, Poribacteria, Planctomycetes).

Interestingly, Southwell et al. [8] found that NO_x_^−^ made up the majority of the DIN pool from *X. muta* and that NO_x_^−^ was almost entirely NO_3_^−^. In this study we observed, in addition to positive net NO_3_^−^ fluxes, a greater net efflux of NH_4_^+^ for all samples of *X. muta*. For some *X. muta* populations (i.e., LSI and LC) the flux of NH_4_^+^ had a significant impact on total DIN fluxes. These differences in the fluxes of NH_4_^+^, probably generated from the utilization of nitrogen rich POM by the sponge host, are unusual given there is an active nitrifying community [7], [8], and a prokaryotic photosynthetic community [22] that could readily utilize NH_4_^+^ in this sponge [54].

The fluxes of DIN from sponges such as *X. muta* can have a significant impact on the availability and composition of DIN on coral reefs [7], [8]. Results of the current study indicate that fluxes of DIN from *X. muta*, the primary contributor to DIN on Caribbean coral reefs [7], [8], is more complex than previously thought and is significantly different between locations. Additionally, these fluxes vary over time as a previous study of the same population of *X. muta* from LSI in 2010 showed both positive and negative fluxes of NO_x_^−^ with a net ΔNO_x_^−^ of −0.27 µM±0.06 (mean ±SE) [Fiore and Lesser unpublished].

Natural abundance stable isotope values have been commonly used to trace sources of C and N through the food chain [55]. Nitrogen fixation yields an average *δ* ^15^N signature of approximately 0.0‰ [56], [57], and trophic enrichment typically results in a +2.2 to +3.5‰ increase per trophic level for *δ* ^15^N [58], [59]. Therefore, several studies have used a cutoff of ≤2.0‰ to indicate N from a fixed source [23], [60], [61]. Additionally, carbon fixation by marine phytoplankton typically results in *δ* ^13^C values of about −19 to −24‰ [55], with an average of +0.5 to +1.0‰ enrichment per trophic level [62]. Based on previous studies that have used stable isotope analysis to investigate the relationship between sponges and their symbionts [6], [7], [49], a cutoff for *δ* ^13^C of −18‰ or lower was used as an indication of photoautotrophic carbon fixation for *X. muta*. The bulk stable isotopic values measured for both C and N in *X. muta* tissue, comprising both the host tissue and prokaryotic biomass, were not significantly different between sites and similar to those documented in previous studies on *X. muta* [22], [23].

Using the cutoff values described above, there is no stable isotopic evidence that nitrogen fixation was occurring in *X. muta* (Fig. 1) although *nif*H genes have been sequenced from *X. muta* [Fiore and Lesser unpublished]. The δ^13^C values of the sponge samples show evidence of photoautotrophy, although we cannot say definitively that there is transfer of C from symbionts to the sponge. Freeman and Thacker [49] demonstrated that high microbial abundance (HMA) sponges, such as *X. muta*, can obtain either C or N, or both, from their symbionts. Any interpretation of tissue stable isotopic signatures must, however, include heterotrophy on picoplankton for sponges. Based on the feeding study of LSI sponges, and despite the high abundance of resident bacteria, sponges are actively and non-selectively filtering most of the bacteria from the ambient water, which would supply significant amounts of POC and PON that could be potentially used by the host which is consistent with studies on other sponge species from the Caribbean [5], [37]. Taken together, the results of this study suggest some site related differences with sponges from the more open ocean sites of LSI and LC being more dependent on photoautotrophic sources of C and coastal FL sponges being more dependent on POM for their C requirements (Fig. 1).

The tracer experiments provide additional insight into what may be occurring in terms of the dynamics of C and N uptake by the prokaryotic community. Results of the HCO_3_^−^ tracer experiment indicate that there is uptake by prokaryotes and then immediate equilibrium of C with the sponge tissue. These results show that there is an active autotrophic community present in *X. muta* and provides evidence for transfer of C from symbiont to host. Uptake of HCO_3_^−^ is not limited to autotrophs, and heterotrophic bacteria may have also taken up HCO_3_^−^ for use in aneplurotic pathways [63].

The NH_4_^+^ tracer experiment indicates that the symbiotic prokaryotic community of *X. muta* actively assimilated the NH_4_^+^ and the host sponge is the likely source of NH_4_^+^. The small increase in sponge host *δ* ^15^N during the NH_4_^+^ experiment may also indicate direct uptake of NH_4_^+^ by the sponge, as has been demonstrated for corals [64]. Alternatively, as in the NO_3_^−^ tracer experiment, the accumulation of tracer in the sponge fraction may also be explained by transfer of N from the bacteria to the host.

NO_3_^−^ can also be utilized by the prokaryotic community, as demonstrated by the increased *δ* ^15^N of the bacterial fractions incubated with ^15^NO_3_^−^ (Fig 4 A). Photosynthetically driven NO_3_^−^ uptake has been demonstrated in planktonic communities [65], and may explain the increase in NO_3_^−^ uptake when the sponges were exposed to natural solar radiation. However, it should be noted that heterotrophic bacteria could take up NO_3_^−^ as well [66]. As the symbiotic nitrifiers in the sponge produce NO_3_^−^ it would provide a source of NO_3_^−^ for uptake by the photosynthetic community as well as a substrate for denitrification. Similarly, NH_4_^+^ is also a likely substrate for aerobic ammonia oxidation by the crenarcheote community, which is supported by the recovery of crenarchaeal *amo*A genes in *X. muta* [34], expression of *amo*A genes [34] and tracer studies [8]. If assimilatory and dissimilatory processes are competing for NO_3_^−^, NO_2_^−^, and NH_4_^+^, which have been documented in other communities [67], then this may have a significant role in nitrogen cycling in the sponge holobiont. Lastly, the anaerobic oxidation of NH_4_^+^ (anammox) is another process that may utilize both NH_4_^+^ and the NO_2_^−^ generated from nitrification. The rates, however, of anammox are relatively low in the water column [68], as is the only documented rate for anammox in sponges [16]. It is possible that anammox may occur within anoxic microhabitats of *X. muta*, and support for this is provided by the presence of planctomycete bacteria in *X. muta* [35,Fiore and Lesser unpublished] but the nutrient flux data clearly show that a net efflux of NH_4_^+^ is still occurring in all sponges suggesting an abundance of this substrate for either nitrification or anammox. Other factors that may be important in explaining the observed variability in net fluxes of DIN include the variability in tissue O_2_ concentration as a result of pumping activity [10] and the concentration of other compounds that are known to influence N cycling such as H_2_S. Variable O_2_ concentrations within the sponge tissue will determine the relative rates of nitrification and denitrification [10], [53], while H_2_S is known to inhibit nitrification and denitrification [69], [70]. H_2_S may be present in *X. muta*, as bacteria involved in sulfur cycling have been recovered from this sponge (Chromatiales, Syntrophobacteraceae, Fiore and Lesser unpublished). If nitrification and denitrification are tightly coupled, then variations in H_2_S or O_2_ concentrations may indeed influence the rates of these processes and the net fluxes of various species of DIN.

**Figure 5:**
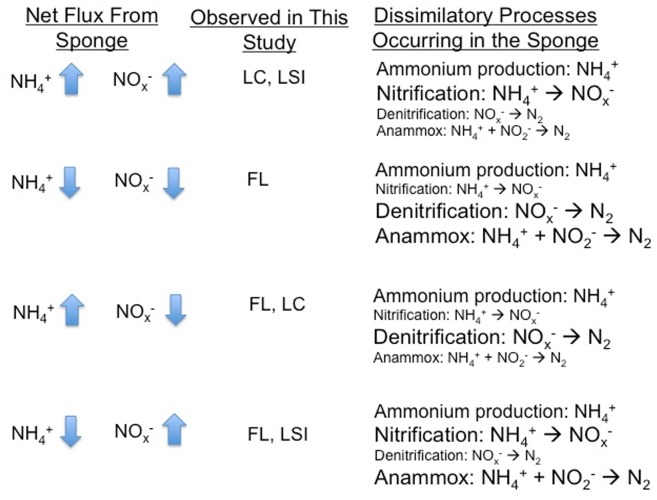
Potential dissimilatory N transformations occurring in *X.* *muta* based on the observed net flux of NH_4_^+^ and NO_x_^−^ from the sponge. Font size for a given process indicates the relative importance of that process. Locations may appear more than once due to differences in individual sponges at each location.

If we consider each outcome in terms of net flux of DIN from *X. muta* from the current study, we can model which dissimilatory processes are likely occurring that can then be used to formulate testable hypotheses (Fig. 5) for future studies. The model also allows us ask questions on the broader ecological impacts of sponge-derived DIN; for example, LC sponges, which generally had a net positive flux of both NH_4_^+^ and NO_x_^−^ (Fig. 5) and often NH_4_^+^ was a significant component to total DIN (Fig. 2), may differentially influence N cycling on the surrounding coral reef relative to FL or LSI sponges. We do not know the extent to which sponge-derived DIN influences the biogeochemistry and ecology of the surrounding habitat, but studies on multi-species sponge assemblages, and coral reef communities dominated by active suspension feeding sponges, have shown the significant role of active suspension feeding and the coupling of POC and PON from the water column to the benthos [71], [72]. The composition of DIN released into the water column by sponges would influence how it might be utilized, and who utilizes it in the surrounding environment, as NH_4_^+^ is more readily incorporated into biomass than NO_3_^−^ which can then potentially support local increases in planktonic community production [73]. As discussed by Southwell et al. [8], excess inorganic nutrients, such as release of DIN by sponges, may have detrimental effects on coral reef ecosystems by stimulating an increase in the growth of fleshy algae in the absence of herbivores [8]. It is important that further research be done to determine the ecosystem level effects of DIN release by sponges, and particularly from *X. muta* in regards to Caribbean coral reefs as it is believed to be a primary contributor of DIN released by sponges [8].

We have shown that the flux of DIN from three populations of *X. muta* is highly variable which may have a significant impact on the availability of DIN on coral reefs given the high abundance of these sponges. Nitrification had been previously demonstrated to occur in *X. muta*, and we show here that other nitrogen transformations including denitrification and/or anammox may occur in these sponges as well as the importance of active suspension feeding on the nitrogen rich pool of picoplankton. Further work is needed to better characterize the flux of DIN from *X. muta* and other sponges on Caribbean coral reefs including whether sponges can fix N_2_. This will require additional investigations on the functional activity of the symbiotic prokaryotic community of sponges using a combination of experimental and molecular approaches (i.e., transcriptomics) that will yield insight into the taxonomy and function of this community, and how this impacts nutrient fluxes and biogeochemical cycling on Caribbean coral reefs.
